# Supplementary material for: Content-rich biological network constructed by mining PubMed abstracts
Source: BMC Bioinformatics. 2004 Oct 8;5:147. doi: 10.1186/1471-2105-5-147 (PMC528731; doi:10.1186/1471-2105-5-147)
Supplement: Additional File 5 — The original Chilibot query results of the term "long-term potentiation (LTP)" and 22 other terms, limiting the latest references analyzed to the years 1990, 1995, 2000, and 2004. [file 1471-2105-5-147-S5.bz2 › chilibotAdditionalFile5/ltp2004/html/TRKB_CREB.html]

 


 **TRKB** and **CREB** 
  
Found 13 abstracts in PubMed,  **13 abstracts were retrieved and analyzed**.  


---

 Search Google  |
 PDF files only 
|  EDU domain only 

---

**Interactive relationship** (e.g. stimulation, inhibition, etc)

- Our results provide genetic evidence that  **TrkB**  mediates hippocampal plasticity via recruitment of PLCgamma, and by subsequent phosphorylation of CaMKIV and  **CREB** .  Ref: 12367511 Neuron, 2002
- These findings indicate that upregulation of  **CREB**  is a common action of chronic antidepressant treatments that may lead to regulation of specific target genes, such as BDNF and  **trkB** , and to the long term effects of these treatments on brain function.  Ref: 8601816 J Neurosci, 1996

**Parallel relationship** (e.g. studied together, co-existance, homology, etc.)

- **CREB**  phosphorylation required  **trkB**  activation because treatment with the trk inhibitor k252a completely blocked  **CREB**  phosphorylation.  Ref: 10751432 J Neurosci, 2000
- The opposite effect was observed in  **trkB**  neurons, suggesting that MAPKs and  **CREB**  act in parallel pathways.  Ref: 12367511 Neuron, 2002
- Molecular Plasticity in the Rat Auditory Brainstem Modulation of Expression and Distribution of Phosphoserine, Phospho  **CREB**  and  **TrkB**  after Noise Trauma.  Ref: 12811001 Audiol Neurootol< MedlineTA>Audiol Neurootol, 2002
- A recent study using mice carrying a mutation in a specific tyrosine residue of  **trkB**  pinpoints a necessary role for the phospholipase Cgamma pathway in  **CREB**  activation and LTP maintenance.  Ref: 12689763 Trends Neurosci, 2003
- The use of a novel microbead injection method in our blocking experiments and Taqman reverse transcription polymerase reaction RT PCR for RNA quantification, have enabled us to evaluate the contribution of different pathways to the exercise induced increases in the mRNA levels of BDNF,  **TrkB** ,  **CREB** , and synapsin I.  Ref: 14622908 Neuroscience, 2003
